# Hollow polydopamine nanoparticles loading with peptide RL-QN15: a new pro-regenerative therapeutic agent for skin wounds

**DOI:** 10.1186/s12951-021-01049-2

**Published:** 2021-10-02

**Authors:** Huiling Sun, Ying Wang, Tiantian He, Dingwei He, Yan Hu, Zhe Fu, Yinglei Wang, Dandan Sun, Junsong Wang, Yixiang Liu, Longjun Shu, Li He, Ziwei Deng, Xinwang Yang

**Affiliations:** 1 Department of Anatomy and Histology & Embryology, Faculty of Basic Medical Science, Kunming Medical University, Kunming, 650500 Yunnan China; 2 Key Laboratory of Applied Surface and Colloid Chemistry, National Ministry of Education, Shaanxi Key Laboratory for Advanced Energy Devices, Shaanxi Engineering Lab for Advanced Energy Technology, School of Materials Science and Engineering, Shaanxi Normal University, Xi’an, 710119 Shaanxi China; 3 Key Laboratory of Chemistry in Ethnic Medicine Resource, State Ethnic Affairs Commission & Ministry of Education, School of Ethno-Medicine and Ethno-Pharmacy, Yunnan Minzu University, Kunming, 650504 Yunnan China; 4 Department of Dermatology, First Affiliated Hospital of Kunming Medical University, Kunming, 650500 Yunnan China

**Keywords:** Hollow polydopamine, Nanoparticles, RL-QN15, Wound healing, Pro-regenerative agent

## Abstract

**Background:**

Although the treatments of skin wounds have greatly improved with the increase in therapeutic methods and agents, available interventions still cannot meet the current clinical needs. Therefore, the development of new pro-regenerative therapies remains urgent. Owing to their unique characteristics, both nanomaterials and peptides have provided novel clues for the development of pro-regenerative agents, however, more efforts were still be awaited and anticipated.

**Results:**

In the current research, Hollow polydopamine (HPDA) nanoparticles were synthesized and HPDA nanoparticles loading with RL-QN15 (HPDAlR) that was an amphibian-derived peptide with obvious prohealing activities were prepared successfully. The characterization, biodistribution and clearance of both HPDA nanoparticles and HPDAlR were evaluated, the loading efficiency of HPDA against RL-QN15 and the slow-releasing rate of RL-QN15 from HPDAlR were also determined. Our results showed that both HPDA nanoparticles and HPDAlR exerted no obvious toxicity against keratinocyte, macrophage and mice, and HPDA nanoparticles showed no prohealing potency in vivo and in vitro. Interestingly, HPDAlR significantly enhanced the ability of RL-QN15 to accelerate the healing of scratch of keratinocytes and selectively modulate the release of healing-involved cytokines from macrophages. More importantly, in comparison with RL-QN15, by evaluating on animal models of full-thickness injured skin wounds in mice and oral ulcers in rats, HPDAlR showed significant increasing in the pro-regenerative potency of 50 and 10 times, respectively. Moreover, HPDAlR also enhanced the prohealing efficiency of peptide RL-QN15 against skin scald in mice and full-thickness injured wounds in swine.

**Conclusions:**

HPDA obviously enhanced the pro-regenerative potency of RL-QN15 in vitro and in vivo, hence HPDAlR exhibited great potential in the development of therapeutics for skin wound healing.

**Graphic Abstract:**

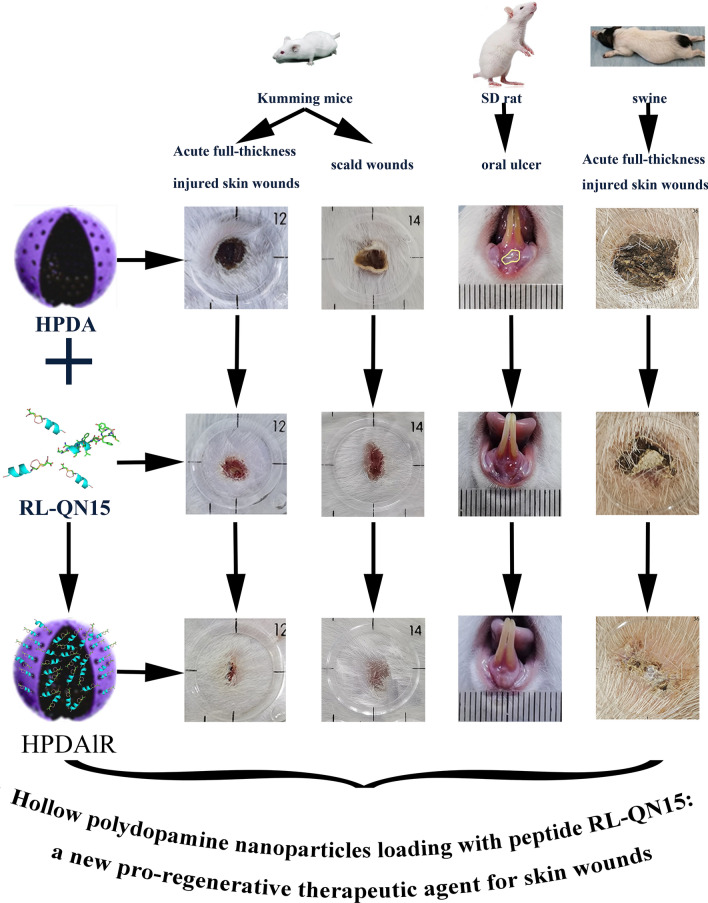

**Supplementary Information:**

The online version contains supplementary material available at 10.1186/s12951-021-01049-2.

## Introduction

As the largest organ of the human body, skin participates in many important physiological functions, such as sweating, fever, and pain perception [1, 2]. Skin is also an important barrier between the internal body and external environment, protecting internal organs and tissues from skin rupture caused by physical, mechanical, and chemical factors and infections caused by pathogenic microorganisms [3]. Once the skin is damaged, a highly complex and dynamic regenerative process, including hemostasis, inflammation, cell proliferation, and tissue reconstruction, starts immediately [4]. However, this process is easily interrupted by a variety of factors, resulting in recalcitrant wounds that can be exploited by pathogens, causing secondary infections, water and electrolyte disorders, septic shock, multi-organ failure, and even death [4]. Therefore, rapid and effective wound healing is critical to human health and survival [5]. In recent years, even many more treatment options have been discovered, such as gene therapy, growth factor therapy, platelet-rich plasma therapy, stem cell-based therapy, and tissue engineering, pro-healing drugs therapy still plays a vital role [6]. Existing wound healing drugs can be divided into two main categories: small molecule compounds derived from plants and proteins represented by epidermal growth factors. However, these drugs also have their own drawbacks. For example, the former is unstable, with poor permeability, solubility, and activity, while the latter are expensive, require strict preservation conditions, and are prone to hypertrophic scarring [7–10]. In short, skin trauma has brought serious material and economic burdens to human society. Thus, exploring ways in which to promote wound healing and developing novel pro-regenerative agents are critical.

Today, cutting-edge nanotechnology offers an unprecedented opportunity for trauma therapy innovation to improve the effectiveness of current medications [11]. Nanomaterials, such as nanoparticles, nanocomposites, hydrogels, and brackets, are used in wound therapy as carriers of therapeutic agents beneficial for wound closure [11–13]. Nanodrug delivery systems can anchor bioactive molecules to the application area to enhance bioavailability, improve solubility, prolong action time, and reduce damage induced by degrading enzymes, thus improving the therapeutic effects [11]. Therapeutic molecules are loaded onto nanoparticles and released continuously after reaching the application area. There are a variety of techniques that can be used to determine the in vitro release patterns after load of therapeutic molecules by nanoparticles [14]. Dopamine is rich in catecholamines and amino groups, which can form polydopamine shells on core material after polymerization, and thus form a variety of core–shell structures. Among them, hollow polydopamine (HPDA) nanoparticles, derived from the core–shell through chemical dissolution [15], have attracted considerable attention due to their low density, high surface area to volume ratio, excellent surface permeability, remarkable loading capacity, and easy-to-control morphology [16–18]. Previous reports have shown that biodegradable iron-coordinated HPDA nanospheres can be used for dihydroartemisinin delivery and tumor cell treatment [19]. However, few reports have explored the application of HPDA nanoparticles in skin wound healing.

Since the first bioactive peptide was artificially synthesized by Robert Bruce Merrifield in 1953, extensive research on peptide drugs has been carried out worldwide [20]. Recent studies have shown that animal and plant peptides exhibit a wide range of biological activities, such as alleviation of hyperuricemia, antibacterial and antioxidative activity [21–25]. At the same time, it is also widely concerned about the promoting repair peptide [26, 27]. However, compared with small molecule drugs, their application is susceptible to physical and chemical states (e.g., pH, enzymes, and temperature) [28, 29]. We previously extracted a short peptide RL-QN15 with amino acid sequence of ‘QNSYADLWCQFHYMC’, from the skin secretions of amphibian *Rana limnocharis* and our results have indicated that RL-QN15 exerted significant prohealing activity both in vivo and in vitro by activating MAPK and Smad signaling pathways to selectively regulating the release of healing-involved cytokines (transforming growth factor-β1 (TGF-β1), tumor necrosis factor-α (TNF-α), interleukin-1β (IL-1β)) from macrophage, hence promoting the migration an proliferation of skin cells, accelerating the formation of granulation and the process of re-epithelialization of skin wounds. RL-QN15 may represent one of the most powerful pro-regenerative agents identified yet in nature and hold promising potential to be a novel pro-regenerative drug candidate [27]. However, although bioactive peptides show strong potential in the promotion of wound healing, these molecules have inherent disadvantages such as easily hydrolyzed by enzyme, short half-life in vivo [30]. Thus, we hope that the potential of RL-QN15 to promote wound tissue regeneration can be improved through some specific ways, more efforts are still needed and awaited.

In the current research, we successfully prepared HPDA nanoparticles that were characterized by Transmission electron microscopy (TEM), Energy-dispersive X-ray spectroscopy (EDX), Fourier transform infrared spectroscopy (FTIR) and X-ray photoelectron spectroscopy (XPS). HPDA nanoparticles loading with RL-QN15 (HPDAlR) was also successfully obtained and characterized by FTIR and XPS. Our results revealed that both HPDA nanoparticles and HPDAlR showed no obvious toxicity, HPDA nanoparticles exerted no prohealing potency in vivo and in vitro. Interestingly, compared with RL-QN15 alone, HPDAlR exerted much more powerful ability to induce the selective release of healing-involved cytokines from macrophage or mice skin wounds, accelerate the healing of scratch of keratinocyte. Importantly, in comparison with RL-QN15, by evaluating on animal models of full-thickness injured skin wounds in mice and oral ulcers in rats, HPDAlR showed significant increasing in the pro-regenerative potency of 50 and 10 times, respectively. Moreover, HPDAlR enhanced the prohealing efficiency of peptide RL-QN15 against skin scald in mice and full-thickness injured wounds in swine. In summary, our results indicated that the in vivo and in vitro pro-regenerative potency of RL-QN15 was significantly enhanced through the loading and slow-releasing efficiency of HPDA nanoparticles and highlighted the strategy using HPDA nanoparticles to load peptide with prohealing activity represented a novel intervention for the skin wounds and thus HPDAlR hold great potential to be promising pro-regenerative therapeutics.

## Results and discussion

### Preparation and properties of HPDA nanoparticles and HPDAlR

Nanoparticles are widely used in modern biomedicine due to their surface functionalization, targeting ability, degradability, and biocompatibility [31–34]. Recently, nanoparticles have attracted attention in the field of wound healing, such as the application of biomimetic elastomeric peptide-based nanofibrous matrices, engineered nanomaterial for infection control and treatment, and nanotechnology for skin wound repair [35–37]. Hollow colloidal particles in nanomaterial are of particular interest due to their low density, excellent surface permeability, remarkable loading capacity, and good morphology [17]. These unique properties make hollow colloidal particles widely applicable in chemical catalysis, biomedicine, optics, electronics, energy storage and conversion, environmental protection, anti-tumor treatment, anti-oxidation, drug transport, and tissue regeneration [16, 38, 39]. HPDA has all the advantages of hollow colloids but is smaller than most nanoparticles. To date, however, research on the application of such important nanoparticles in promoting skin wound regeneration remains limited.

**Fig. 1:**
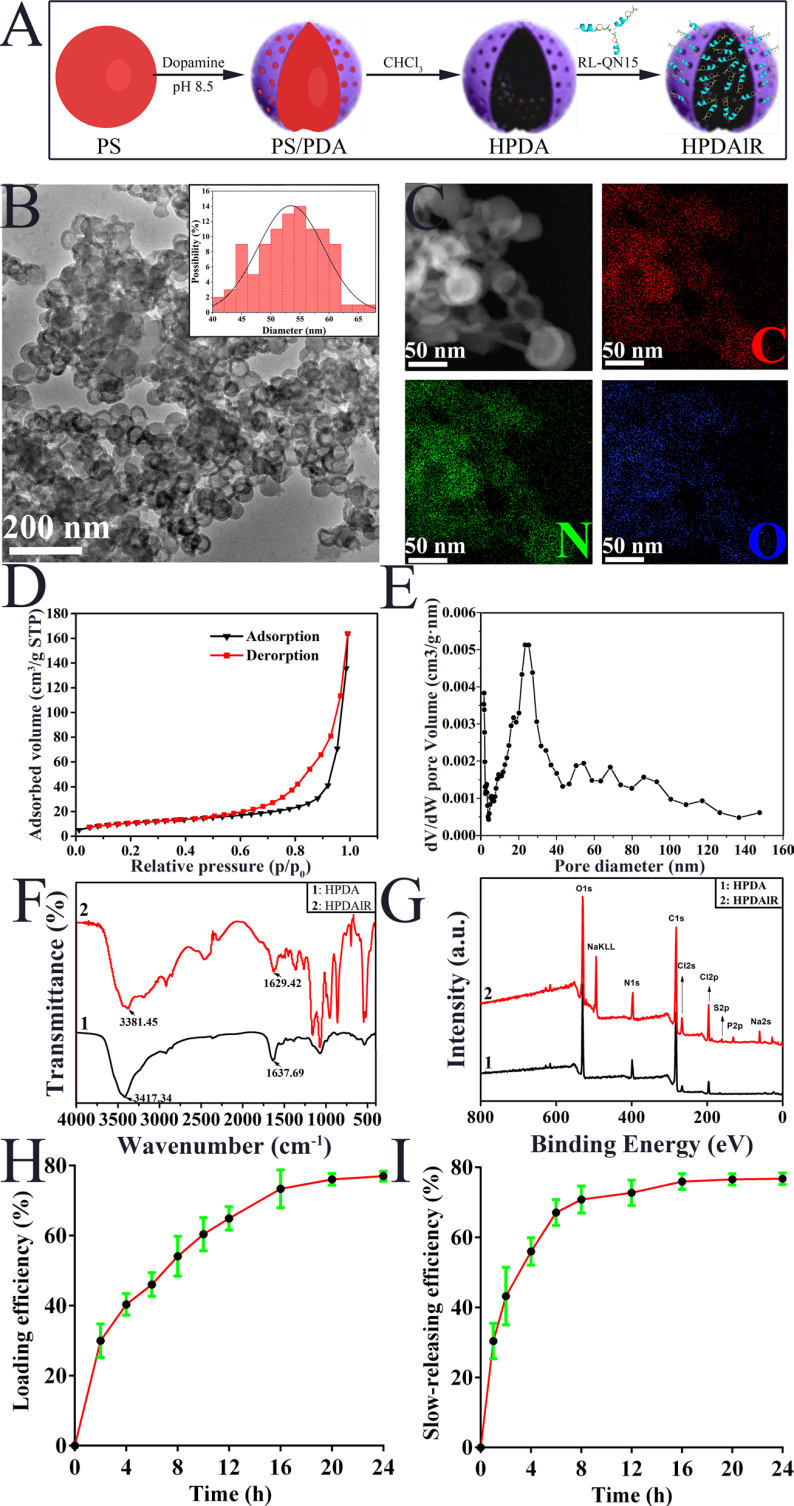
Characterization of HPDA nanoparticles and HPDAlR. **A** The scheme detailing the preparing procedure of HPDA and HPDAlR. PS powder was thoroughly mixed with dopamine aqueous solution at room temperature, with PS/PDA composite nanospheres then formed after stirring using a magnetic agitator. These composite nanospheres were centrifuged, washed and dried. The organic template was removed to obtain HPDA nanospheres. The RL-QN15 peptide was incubated with the decentralized HPDA nanoparticles, then the mixture was centrifuged and the supernatants were discarded and the rests were considered as HPDAlR. **B** TEM image of HPDA nanoparticles. HPDA particles with an average diameter of 52 nm showed well-defined spherical morphologies and hollow structures. Scale bar (200 nm) was indicated by a line in the bottom-left corner of Figure. **C** EDX analysis of HPDA nanoparticles revealed a uniform distribution of C, N, and O elements, which implied the formation of HPDA nanoparticles. The scale bar (50 nm) was indicated by lines in the bottom-left corner of in Figures. **D** Nitrogen adsorption–desorption isotherms of HPDA nanoparticles. **E** Pore size distribution of HPDA nanoparticles. **F** FTIR analysis of HPDA (black line) and HPDAlR (red line). By the loading of RL-QN15, peaks at 3 417.34 cm^−1^ and 1 637.69 of HPDA were shifted to 3381.45 cm^−1^ and 1629.42 cm^−1^. **G** XPS analysis of HPDA (black line) and HPDAlR (red line). After the loading of RL-QN15, compared with the HPDA, a weak S2_P_ signal peak appeared in the HPDAlR sphere spectrum. **H** Loading efficiency of HPDA against RL-QN15. The data were displayed as Mean ± SD, n = 4. **I** Slow-releasing efficiency of HPDAlR against RL-QN15. The data were displayed as Mean ± SD, n = 4

In this study, HPDA nanoparticles were synthesized as per earlier research (Fig. 1A) [40]. TEM images (Fig. 1B) confirmed that the resultant HPDA nanoparticles showed well-defined spherical morphologies and hollow structures. Their average diameter was 52 nm (Fig. 1B). The elemental mapping patterns revealed a uniform distribution of C, N, and O elements (Fig. 1C), further confirming the formation of HPDA nanoparticles. Brunauer–Emmett–Teller (BET) was used to analyze surface physical characteristics of HPDA nanoparticles, as shown in Fig. 1D and E, the nitrogen adsorption–desorption isotherms of HPDA demonstrated hysteresis loops, which is characteristic of mesoporous materials. Besides, as listed in Additional file 1: Table S1, the prepared HPDA had a surface area of 39.1667 m^2^/g, pore volume of 0.252455 cm^3^/g, and pore diameter of 27.2621 nm. Due to their hollow structure and nanoscale spherical morphology, HPDA nanoparticles has been considered as drug carriers to ensure that drug molecules exert satisfactory therapeutic effect [41].

Wound healing involves a series of highly complicated physiological processes, including changes in capillary permeability, cell migration, fibroblast proliferation, endothelial and epithelial cell damage, and dynamic balance between cells, collagen, and capillaries [42]. Peptides that have positive effects on inflammation, proliferation, and remodeling are theoretically healing promoters, e.g., Bv8, Bombesin, and endothelial growth factor (EGF) and vascular EGF-releasing peptides [42, 43]. With the development of biotechnology and peptide synthesis, an increasing number of peptide drugs have been developed and applied clinically [44]. Amphibian skin secretes a variety of bioactive peptides with therapeutic effects, which have been extensively studied. As one of the shortest peptides, RL-QN15 has relatively low synthesis costs but significant ability to promote the healing of skin wounds [27].

Considering the advantages of HPDA nanoparticles, it might be reasonable to speculate that the prohealing potency of RL-QN15 might be enhanced by the loading and slow-releasing of HPDA nanoparticles, hence, as schemed in Fig. 1A, we successfully prepared HPDAlR. Because the FTIR spectra of bioactive substances differ from each other [45], we carried out FTIR qualitative analysis of HPDA and HPDAlR, to verify whether RL-QN15 was successfully loaded onto the HPDA shell. The two curves in Fig. 1F showed the characteristic peaks of HPDA and HPDAlR, respectively. In the HPDA spectrum, the peak at 3 417.34 cm^−1^ was identified as the characteristic absorption peak of N–H and O–H group stretching vibration, while the peak at 1 637.69 cm^−1^ was the characteristic absorption peak of C-O stretching vibration [46]. Compared with HPDA, the peak values of HPDAlR at these two sites were slightly lower, which was due to the existence of an intermolecular hydrogen bond between HPDA and RL-QN15. In addition, HPDA and HPDAlR exhibited different spectral fingerprint regions (1 800–500 cm^−1^). The HPDAlR showed characteristic absorption peaks at 1 629.42 cm^−1^, 1 359.70 cm^−1^, 1 072.40 cm^−1^, 861.11 cm^−1^, 545.10 cm^−1^, and 520.74 cm^−1^. At 1 637.69 cm^−1^, 1 071.70 cm^−1^, and 534.72 cm^−1^, HPDA had stronger absorption peaks than HPDAlR. These results indicated that RL-QN15 was successfully loaded onto the HPDA shell to form HPDAlR. X-ray photoelectron spectroscopy (XPS) was also used to analyze the surface chemical composition of the samples. As shown in Fig. 1G, strong O1s and C1s signal peaks accompanied by weak N1s signal peaks were observed in the HPDA sphere spectrum. After RL-QN15 was loaded onto HPDA, a weak S2p signal peak appeared. These results provide solid evidence that RL-QN15 containing intramolecular disulfide bonds was loaded into the HPDA nanoparticles. In summary, the HPDAlR was successfully prepared for the first time, which was verified by both FTIR and XPS.

The loading and slow-releasing efficacy of HPDA against RL-QN15 was further determined. As shown in Fig. 1H, the loading efficacy of the HPDA against RL-QN15 showed a sharp increase at 2 h after the incubation, then a slight ascending trend along with the elapse of time and achieved a maximum value of 76.97% at 24 h. As illustrated in Fig. 1I, when dispersed in phosphate buffered saline (PBS), HPDAlR itself started to release RL-QN15 into PBS in a slow-releasing manner. The HPDAlR released almost half of the RL-QN15 peptide in ~ 4 h and reached a release peak of 75.95% at 16 h. Peptides are fragile and easily degraded by various endogenous and exogenous enzymes [44]. Therefore, coating peptides with nanoparticles could not only help to reduce the degradation induced by various enzymes on the skin surface but also sustain an effective concentration, and hence the increase in the prohealing activities of RL-QN15 might be anticipated.

### The toxicity of HPDA and HPDAlR against human keratinocyte, mouse macrophage and mice

**Fig. 2:**
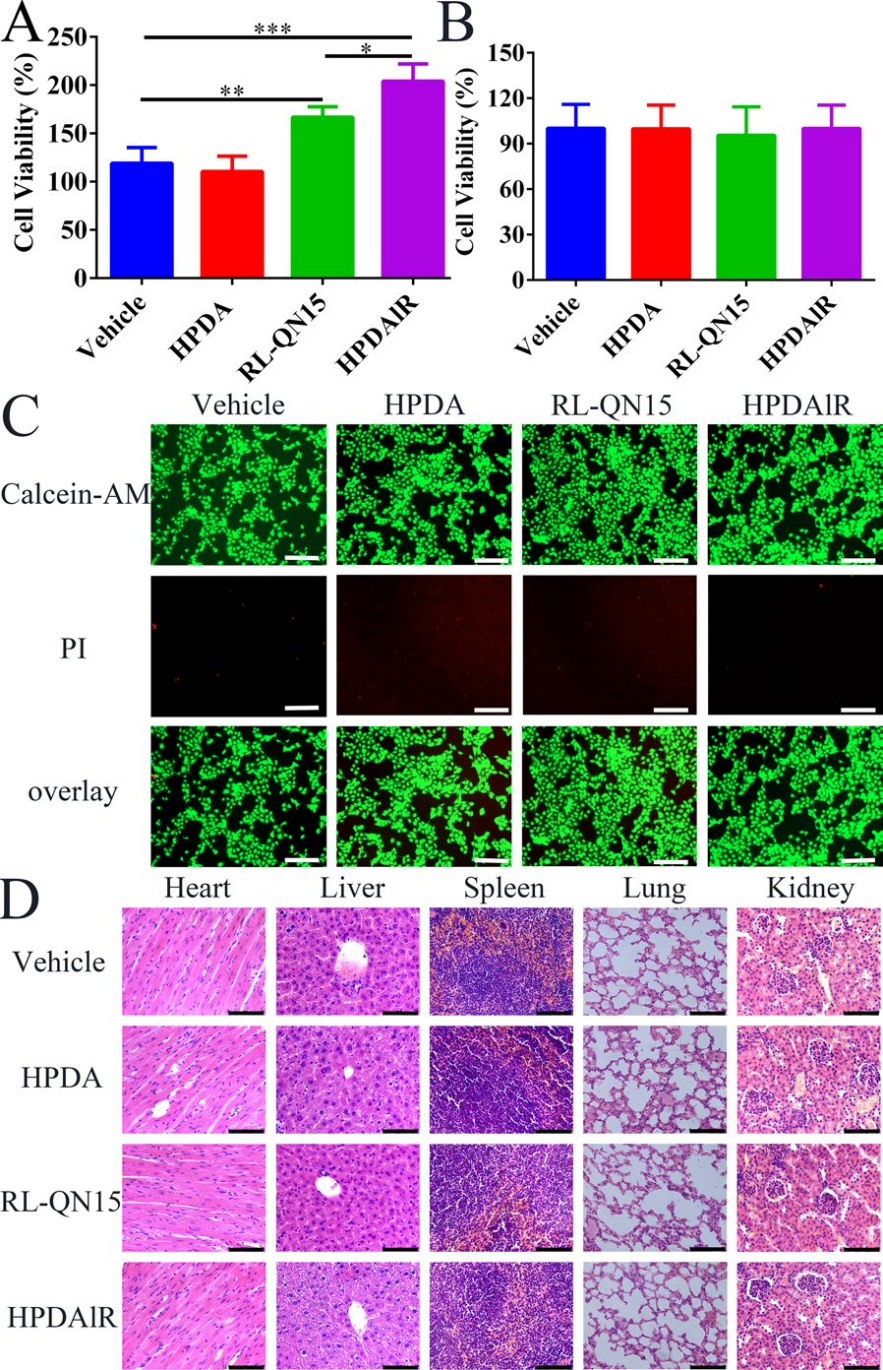
The toxicity of HPDA and HPDAlR.** A** Relative viabilities of HaCaT cells treated with vehicle (serum-free medium), HPDA (0.01 mg/mL), RL-QN15 (1 nM) and HPDA (0.01 mg/mL)lR(1 nM). Data were expressed as Mean ± SD of three independent experiments performed in quadruplicate. *P < 0.05, **P < 0.01 and ***P < 0.001 indicated statistically significant differences between the two groups (Student *t*-test). **B** Relative viabilities of RAW264.7 cells treated with vehicle (serum-free medium), HPDA (0.01 mg/mL), RL-QN15 (1 nM) and HPDA (0.01 mg/mL)lR(1 nM). Data were expressed as Mean ± SD of three independent experiments performed in quadruplicate. **C** The representative confocal fluorescence images of HaCaT treated with vehicle (serum-free medium), HPDA (0.01 mg/mL), RL-QN15 (1 nM) and HPDA (0.01 mg/mL)lR(1 nM). The live/cells were stained by Calcein-AM with green fluorescence and PI with red fluorescence, respectively. Scale bar: 75 µm. **D** H&E staining of the main organs from mice of whose dorsal skin wounds treated with vehicle (PBS), HPDA (0.1 mg/mL), RL-QN15 (1 nM), and HPDA (0.1 mg/mL)lR(1 nM). Scale bar: 100 µm

The toxic evaluation should be initially performed before the determination of biological activities of HPDA and HPDAlR. As shown in Fig. 2A, HPDA (0.01 mg/mL) had no obvious effect on the viability of keratinocyte HaCaT, and RL-QN15 (1 nM) could significantly increase the viability of HaCaT, which was consistent with the results from our previous research [27]. Interestingly, by the loading of RL-QN15, HPDA (0.01 mg/mL) lR (1 nM) obvious enhanced the viability-promoting ability of RL-QN15 (1 nM) by 37.14 ± 17.41% (n = 4). As displayed in Fig. 2B, HPDA, RL-QN15 and HPDAlR exerted no influence on the viability of mouse macrophage RAW264.7. We further confirmed the positive effects of RL-QN15 and HPDAlR on HaCaT by Live/Dead Cell Viability assay, as displayed in Fig. 2C, almost all of the keratinocyte (HaCaT) cells were stained with Calcein acetoxymethyl ester (Calcein-AM, green fluorescence) and dead cells stained with propidium iodide (PI, red fluorescence) were rarely observed. These results suggested that HPDA and HPDAlR were not cytotoxic against HaCaT and Raw 264.7. More importantly, the topical application of HPDA and HPDAlR to the dorsal skin wounds caused no any death of mice (date not shown), compared with un-treated mice, main organs, including heart, liver, spleen, lung and kidney, showed no obvious histopathological abnormalities (Fig. 2D). Previous studies had certified that the developed HPDA nanoparticles have negligible cytotoxicity [47]. These results are well consistent with the results in this research, the negligible cytotoxicity laid solid foundation for us to investigate the pro-regenerative potency of HPDAlR in vitro and in vivo.

### HPDA enhanced the pro-healing activity of RL-QN15 against cell scratch of keratinocyte

**Fig. 3:**
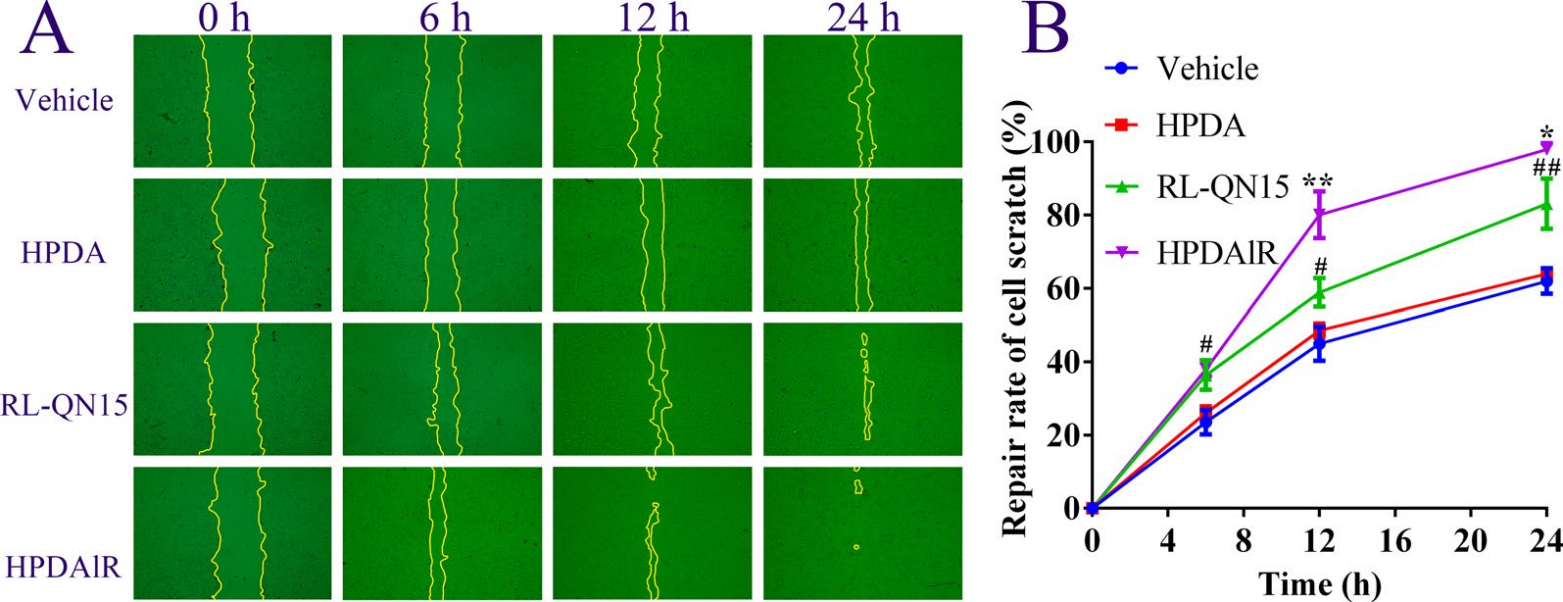
Effects of HPDAlR on the healing of HaCaT scratch.** A** Representative images displaying healing effects of vehicle (PBS), HPDA (0.1 mg/mL), RL-QN15 (1 nM), and HPDA (0.1 mg/mL)lR(1 nM) on healing of HaCaT scratches. **B** Quantitative curves showing the scratch pro-healing effect of HPDAlR was better than RL-QN15. Data were expressed as Mean ± SD of three independent experiments performed in triplicate. ^#^*P* < 0.05 and ^##^*P* < 0.01 indicated statistically significant differences compared to vehicle. ^*^P < 0.05 and ^**^P < 0.01 indicated statistically significant differences compared to RL-QN15 (Student *t*-test)

Keratinocytes plays vital roles in the healing process of skin wounds for their migration fleetly to wound areas and proliferation to promote re-epithelialization of the wound [26]. RL-QN15 (1 nM and 10 nM) has been proved to significantly promote the healing of HaCaT scratch with a pro-healing rate of ≈80% and 95%, respectively [27]. The results that HPDAlR exerted better ability to promote the proliferation than RL-QN15 (Fig. 2A) indicated that HPDA might enhance the promoting effect of RL-QN15 against cell scratch of keratinocyte. As shown in Fig. 3A, when at 24 h, compared with vehicle (PBS), HPDA (0.01 mg/mL) itself didn’t, but RL-QN15 (1 nM) and HPDA (0.01 mg/mL)lR(1 nM) did promote the healing of HaCaT scratch. As quantified in Fig. 3B, the prohealing activities of RL-QN15 and HPDAlR were both time-dependent. The healing rate of RL-QN15 group was 58.89 ± 4.30% (n = 3) at 12 h and 83.07 ± 7.53% (n = 3) at 24 h, whereas the HPDAlR healing rate was 83.07 ± 6.51% (n = 3) at 12 h and 97.95 ± 1.84% (n = 3) at 24 h. Compared with our previous research, we found that the cellular pro-healing effect of HPDA (0.01 mg/mL)lR(1 nM) was equivalent to that of RL-QN15 at 10 nM. All these results indicated that even HPDA nanoparticle itself showed no prohealing activity, by the loading and slow-releasing of HPDA, the cellular prohealing potency of RL-QN15 was significantly enhanced.

### HPDAlR raised the selective modulatory ability of RL-QN15 to induce the release of cytokines from macrophage

**Fig. 4:**
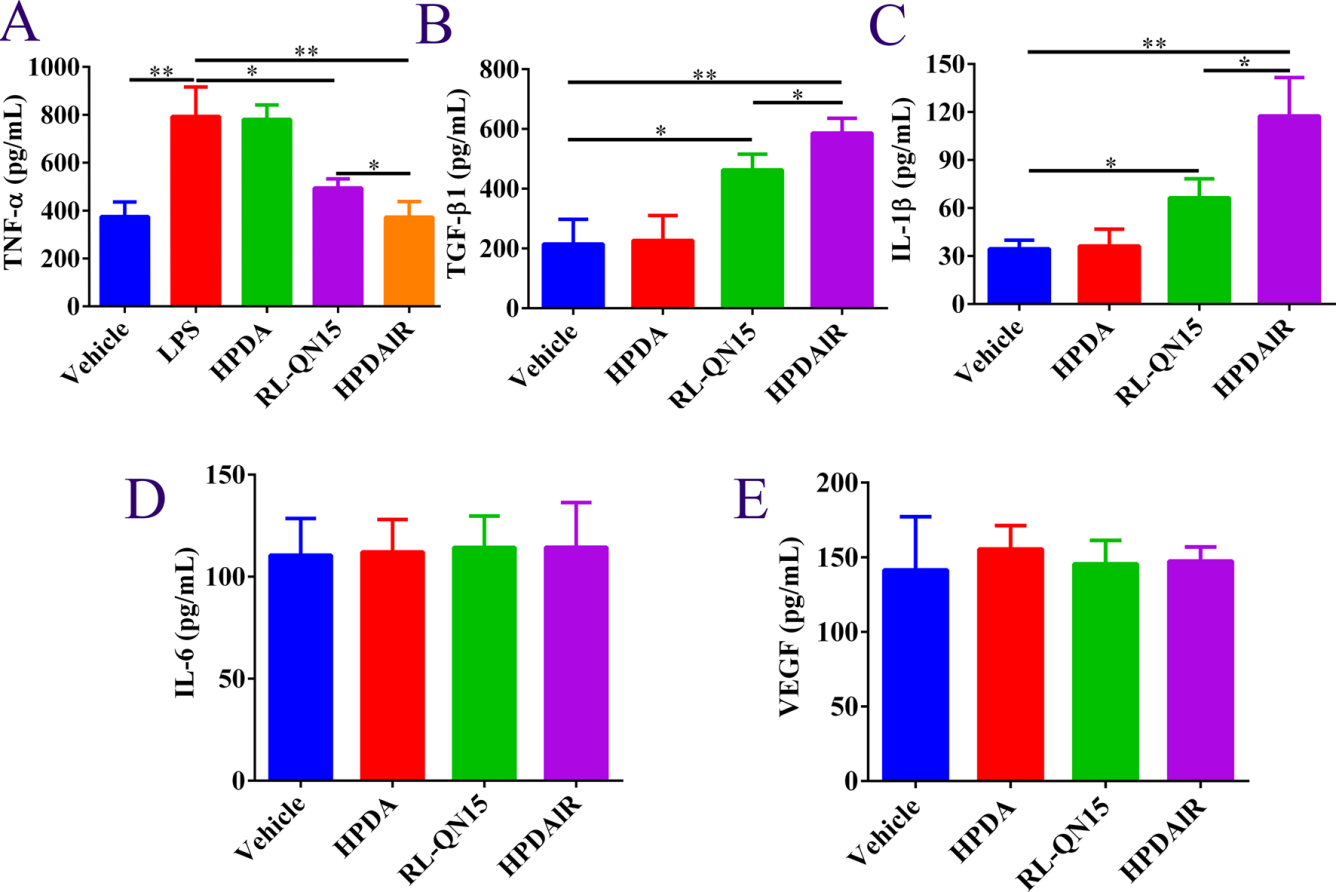
The effects of HPDAlR on the release of cytokines from macrophage.** A**–**E** Effects of HPDAlR on release of TNF-α, TGF-β1, IL-1β, IL-6, and VEGF from RAW264.7 cell respectively. Data were expressed as Mean ± SD of three independent experiments performed in triplicate. *P < 0.05 and **P < 0.01 indicated statistically significant differences between the two groups (Student *t*-test)

Tissue damage induces a complex series of reactions in which macrophages clear cell fragments, activates and eliminate inflammation and promotes tissue fibrosis by releasing cytokines, such as TNF-α, TGF-β1, IL-1β, IL-6 and vascular endothelial growth factor (VEGF) [48]. TNF-α activates neutrophils and lymphocytes to increase the permeability of blood vessel endothelial cells and promote the synthesis and release of other cytokines. TGF-β1 induces macrophages to migrate to the wound sites to promote the proliferation of fibroblasts and the synthesis of cell matrix, and promote the proliferation of epidermal cells. The IL-1β and IL-6 recruit inflammatory cells to secrete pro-healing growth factors to the wound sites [27]. VEGF increase the vascular permeability, proliferation and migration of vascular endothelial cell, and angiogenesis [49]. Our previous study has shown that RL-QN15 significantly blocks the release of lipopolysaccharide (LPS)-induced pro-inflammatory factor TNF-α, induces the release of pro-healing factor TGF-β1 and pro-inflammatory factor IL-1β, but does not induce the release of IL-6 and VEGF [27]. In this research, we found that HPDAlR also played the same role by enhancing the ability of RL-QN15 to selectively regulate the release of cytokines. However, HPDA itself has no effect on the release of these factors from macrophages, as shown in Fig. 4A, compared with LPS-stimulated group, HPDA (0.01 mg/mL) had no effect on the release of LPS-induced TNF-α (793.31 ± 137.65 pg/mL *vs* 780.70 ± 62.80 pg/mL, n = 3). RL-QN15 (1 nM) inhibited the release of TNF-α (495.03 ± 39.25 pg/mL, n = 3), and HPDA (0.01 mg/mL)lR(1 nM) had more significantly inhibiting effects on the release of TNF-α (372.80 ± 73.73 pg/mL, n = 3). In Fig. 4B and C, compared with vehicle and HPDA (0.01 mg/mL), RL-QN15 (1 nM) promoted the release of TGF-β1 and IL-1β, and HPDA (0.01 mg/mL)lR(1 nM) had more significant effects on the release of TGF-β1 and IL-1β. On the RL-QN15 group, the release of TGF-β1 and factor IL-1β were 463.14 ± 58.86 pg/mL and 66.44 ± 13.28 pg/mL (n = 3). However, On the HPDAlR group, the release of TGF-β1 and IL-1β were 586.03 ± 56.48 pg/mL and 117.62 ± 27.17 pg/mL (n = 3).

### HPDA enhanced the pro-regenerative activity of RL-QN15 on acute full-thickness injured skin wounds in mice

**Fig. 5:**
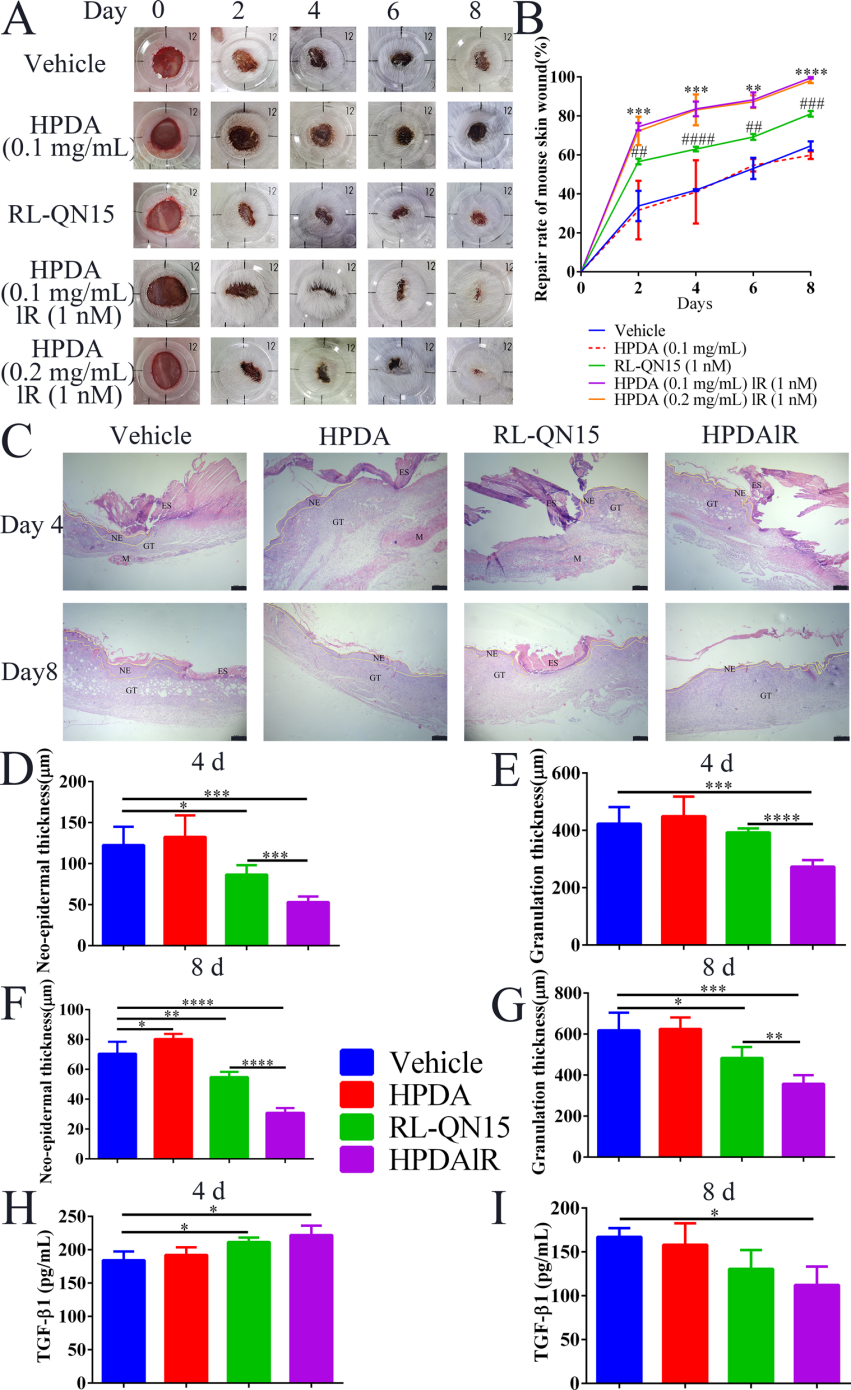
HPDAlR showed much more potent pro-regenerative activity than RL-QN15 against acute full-thickness injured skin wounds in mice.** A** Typical skin wound images of mouse topically treated with vehicle (PBS), HPDA, RL-QN15, HPDA (0.1 mg/mL)lR(1 nM) or HPDA (0.2 mg/mL)lR(1 nM) on postoperative days 0, 2, 4, 6, and 8. **B** Quantitative curves of pro-healing activities of Vehicle (PBS), HPDA, RL-QN15, HPDA (0.1 mg/mL) lR (1 nM), HPDA (0.2 mg/mL)lR(1 nM) groups. Error bars represented SD. ^##^*P* < 0.01, ^###^*P* < 0.001and ^####^*P* < 0.0001 indicated statistically significant differences compared to vehicle. ^**^P < 0.01, ^***^P < 0.001 and ^****^P < 0.0001 indicated statistically significant differences compared to RL-QN15 (Student *t*-test). **C** Histopathological examination of excisional wounds stained with H&E. NE, Neoepidermis; GT, Granulation tissue; ES, Eschar; M, Muscle; Scale bar: 200 μm. **D** and **F.** Quantitative analysis of neo-epidermal thickness on postoperative days 4 and 8. **E** and **G** Quantitative analysis of granulation thickness on postoperative days 4 and 8, respectively. Data were Mean ± SD (n = 9), error bars represent SD. *P < 0.05, **P < 0.01, ***P < 0.001, and ****P < 0.0001 indicated statistical differences between two groups (Student *t*-test). **H** and **I** Effects of local HPDAlR treatment on TGF-β1 content in skin wounds on post-operation day 4 and day 8. Data were Mean ± SD from three independent experiments performed in triplicate. *P < 0.05 indicated statistical differences between two groups (Student *t*-test)

On the basis that HPDAlR significantly enhanced the abilities of RL-QN15 to promote the healing of HaCaT scratch and selectively regulate the release of healing-involved cytokines from macrophage, it might be reasonable to raise the speculation that the in vivo pro-regenerative of RL-QN15 could be up-graded by HPDA. Hence, we topically applied vehicle (PBS), HPDA (0.1 mg/mL), RL-QN15 (1 nM), HPDA (0.2 mg/mL)lR(1 nM), HPDA (0.1 mg/mL)lR(1 nM) to skin wounds in mice twice a day. As shown in Fig. 5A and B, compared with vehicle, HPDA (0.1 mg/mL) itself had no effect on skin wound healing, as contrary, both RL-QN15 (1 nM), HPDA (0.2 mg/mL)lR(1 nM) and HPDA (0.1 mg/mL)lR(1 nM) had significant effects on skin wound healing. On postoperative day 8, the wound healing rates in the vehicle and HPDA groups were 64.60 ± 2.67% and 59.82 ± 2.23% (n = 9), respectively. The healing rate in the RL-QN15 group was 81.05 ± 1.68% (n = 9), which was 1.25 and 1.35 times higher than that of the vehicle and HPDA groups, respectively. More importantly, by loading of HPDA, RL-QN15 achieved a much greater repair effect. On postoperative days 2, 4, 6, and 8, the wound healing rates of RL-QN15 were 56.57 ± 1.65%, 62.99 ± 1.33%, 69.19 ± 1.62%, and 81.05 ± 1.68% (n = 9), respectively. Interestingly, the healing rates of HPDA (0.2 mg/mL)lR(1 nM) increased to 72.29 ± 7.87%, 83.09 ± 9.04%, 87.30 ± 3.72%, and 98.23 ± 1.54% (n = 9), respectively, and the healing rates of HPDA (0.1 mg/mL)lR(1 nM) increased to 74.46 ± 2.19%, 83.58 ± 3.78%, 88.23 ± 4.41%, and 99.52 ± 0.44% (n = 9), respectively. In addition, there was no obvious difference in the prohealing potency pf HPDA (0.2 mg/mL)lR(1 nM) and HPDA (0.1 mg/mL)lR(1 nM), therefore, the concentration of HPDA used in the following experiments was 0.1 mg/mL and HPDAlR represented HPDA (0.1 mg/mL)lR (1 nM). One point should be observed was that, HPDAlR showed an equivalent prohealing activities with RL-QN15 (50 nM) that reported in our previous research [27], hence, it might be reasonable to speculate that by the loading of HPDA nanoparticles and slow-releasing efficiency of HPDAlR, the pro-regenerative potency of RL-QN15 against acute full-thickness injured skin wounds had an markedly increase of ≈ 50 times.

Hematoxylin–eosin (H&E) staining was performed after sampling on postoperative days 4 and 8 (Fig. 5C). And the results were further quantified (Fig. 5D–G). With the passage of time, new epidermis and granulation tissues were gradually formed in each group, but after the application of RL-QN15 and HPDAlR, the regeneration and reconstruction of epidermis and granulation tissues were significantly enhanced. In particular, the HPDAlR group had recovered to normal skin level on the 8^th^ day. On postoperative days 4 and 8, the epidermal thickness of the vehicle and HPDA groups was ~ 130 μm and ~ 80 μm, and the dermal thickness was ~ 450 μm and ~ 650 μm (n = 9), respectively. In the RL-QN15 group, the epidermal thickness was ~ 90 μm and ~ 50 μm, and the dermal thickness was ~ 400 μm and ~ 500 μm (n = 9), respectively. However, in the HPDAlR group, epidermal thickness was 53.10 ± 10.50 μm and 30.87 ± 4.63 μm, and dermal thickness of was ~ 280 μm and ~ 350 μm (n = 9), which were closest to the thickness of normal skin tissue among the four groups.

In addition, previous studies have confirmed the TGF-β1 is the key factor in the healing process of skin wounds and our research has indicated that RL-QN15 induces the release of TGF-β1 from macrophage [27], which was also verified to be enhanced by HPDAlR (Fig. 4B). As shown in Fig. 5H and I, after local application of RL-QN15 (1 nM) and HPDA (0.1 mg/mL)lR(1 nM), the expression of TGF-β1 in wound tissue samples increased significantly at the early stage of wound healing (4^th^ day after operation). Briefly, the TGF-β1 content was 184.37 ± 14.28 (n = 3) pg/mL in the vehicle group, 192.27 ± 13.02 pg/mL (n = 3) in HPDA group, 211.57 ± 7.76 pg/mL (n = 3) in RL-QN15 group, and 221.88 ± 16.19 pg/mL (n = 3) in HPDAlR group, respectively. However, in the late stage of wound repair (postoperative day 8^th^), compared with the vehicle group, HPDAlR colud obviously inhibited the release of TGF-β1 (167.08 ± 11.16 pg/mL *vs* 112.22 ± 24.21 pg/mL, n = 3) in wound tissue. TGF-β1 is an important symbol of regenerating epithelium and an important and indispensable factor of skin matrix and granulation tissue formation. In the early stage of wound healing, the rich level of TGF-β1 ensures the acceleration of the healing of skin wounds. In addition, the TGF-β1 also promotes fibroblasts chemotaxis and the fibrosis, inhibits collagen degradation [50]. Therefore, reducing TGF-β1 in the later stage of wound healing can alleviate the occurrence of fibrosis, thus the formation of the skin scar. So, HPDAlR might accelerate the healing of skin wounds on mice without or with less the formation of scar by dynamically regulating the contents of TGF-β1.

Next, in order to directly observe the biodistribution and clearance of HPDA and HPDAlR, we successfully prepared ICG-labeled HPDA and HPDAlR, which was topically applied to the skin wounds on mice. By tracking the fluorescence of ICG labeled samples using the IVIS® Spectrum in vivo optical imaging system at 0.5, 2, 4, 8, 12, 24, 48 h, as shown in Additional file 1: Figure S1A, it was found that ICG-labeled HPDA and ICG-labeled HPDAlR almost distributed to the whole wounds area. Notably, the remaining ICG-labeled HPDA and ICG-labeled HPDAlR nanoparticles in the whole wounds area rapidly decayed over time, indicating that ICG-labeled HPDA and ICG-labeled HPDAlR nanoparticles might be removed from the skin metabolism. Due to their small size, HPDA still had chance to enter into the blood, so the distribution and clearance of ICG-labeled HPDA and HPDAlR administrated by intraperitoneal injection to mice were also observed. As shown in Additional file 1: Figure S1B and C, after intraperitoneal injection, the sample rapidly distributed to the entire intraperitoneal area. Abdomen images of mice were taken and results showed that 4 h after the injection, both ICG-labeled HPDA and HPDAlR were mainly distributed in the abdominal cavity of the mice, particularly the liver and kidneys. Then, the fluorescence intensity of ICG-labeled HPDA and HPDAlR nanoparticles gradually decayed with time, indicating that ICG-labeled HPDA and ICG-labeled HPDAlR nanoparticles might be removed from the liver and kidneys metabolism.

### HPDA raised the pro-healing activity of RL-QN15 on scald wounds in mice

**Fig. 6:**
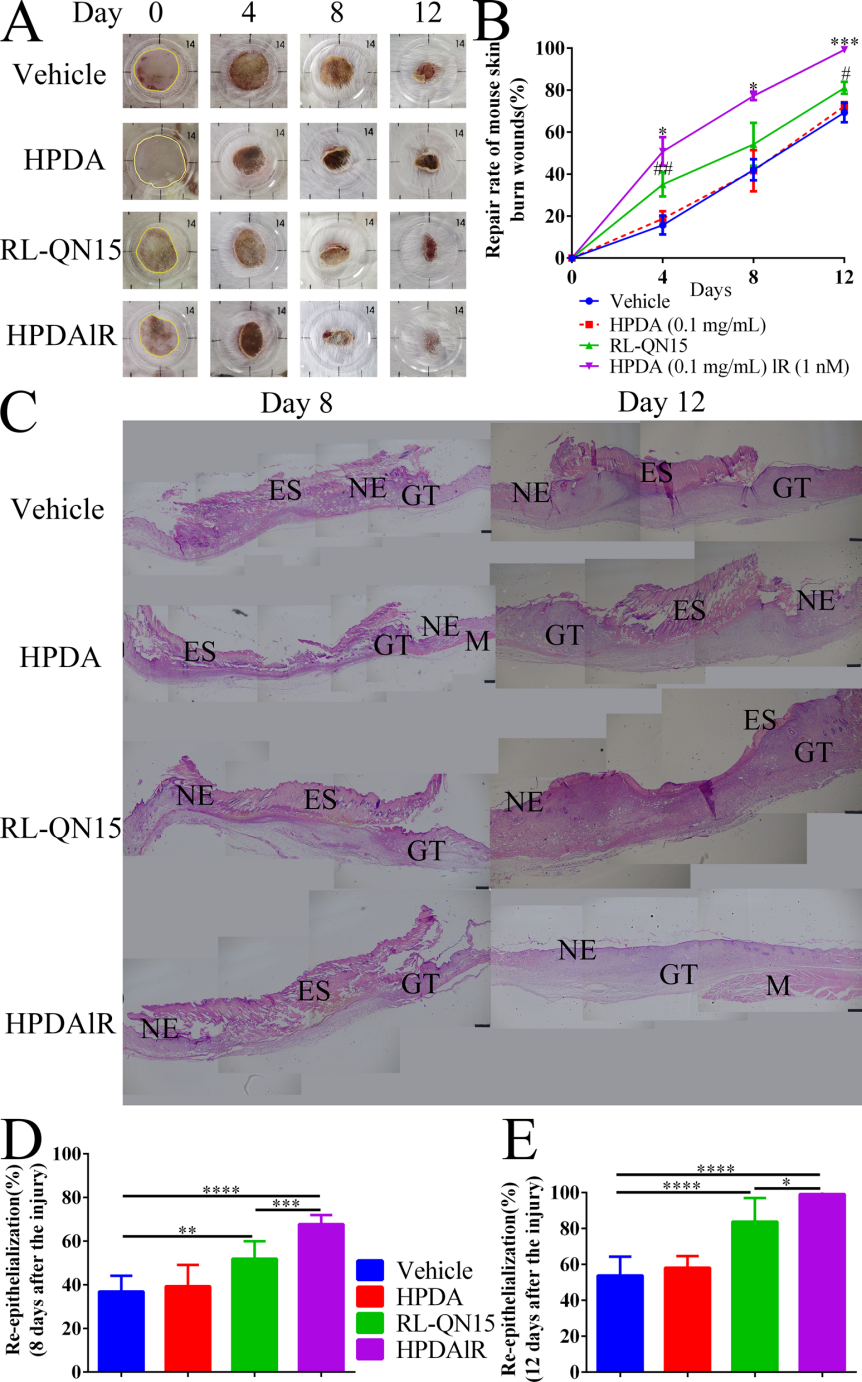
HPDAlR showed much more potent prohealing activity than RL-QN15 against skin scald in mice.** A** Representative images of skin scalds on postoperative days 1, 4, 8, and 12 treated with vehicle (PBS), HPDA (0.1 mg/mL), RL-QN15 (1 nM), and HPDAlR. **B** Quantitative healing rate curves of skin wounds treated with vehicle (PBS), HPDA (0.1 mg/mL), RL-QN15 (1 nM), and HPDAlR. Data were Mean ± SD from nine mice. ^#^*P* < 0.05, and ^##^*P* < 0.01 indicated statistically significant differences compared to vehicle. ^*^P < 0.05, and ^***^P < 0.001 indicated statistically significant differences compared to RL-QN15 (Student *t*-test). **C** Representative images of histomorphological changes in skin scald wounds on postoperative days 8 and 12. Scalded skin sections were stained with H&E. NE: Neoepidermis; GT: Granular tissue, ES: Eschar, M: Muscle. Scale bar: 250 μm. **D** and **E** Quantitative assessment of histological re-epithelialization of epidermis on postoperative days 8 and 12. Data were Mean ± SD from nine mice. *P < 0.05, **P < 0.01, ***P < 0.001, and ****P < 0.0001 indicated statistical differences between two groups (Student *t*-test)

A skin scald mouse model was also employed to evaluate whether HPDA loading could improve the pro-healing activity of RL-QN15. Here, vehicle (PBS), HPDA, RL-QN15, HPDAlR were topically applied to treat scald on the dorsal skins of mice twice a day. As shown in Fig. 6A, the scalded skin injury progressed from waxy white to brown to scab peeling off. On day 12, skin scabs in the vehicle and HPDA groups were the largest, and HPDA nanoparticles showed no positive effect against the healing of scald. Followed by the RL-QN15 group, while those in the HPDAlR group had mostly fallen off. On postoperative day 12, the repair rates in the vehicle and HPDA groups were 69.35 ± 5.30% and 72.41 ± 1.89% (n = 9), respectively, while that in the RL-QN15 group was increased to 81.10 ± 3.05% (1.67 and 1.12 times higher than the vehicle and HPDA groups, respectively). Notably, HPDAlR almost completely healed the scald wound, with an average repair rate of 99.24 ± 0.92% (n = 9), almost 1.22 times that of the RL-QN15 group (Fig. 6B).

We also sampled scalded skin of mice for H&E staining on postoperative days 8 and 12 (Fig. 6C). On postoperative day 8, all experimental groups were still in the inflammatory phase, but new epithelial tissue and granulation tissue appeared in the HPDAlR group. On postoperative day 12, epidermal regeneration and reconstruction were almost finished in the HPDAlR group, and the epidermal status was consistent with that of normal mice. As shown in Fig. 6D and E, in the process of wound healing, the level of re-epithelialization in the HPDAlR group (99.21 ± 1.41%, n = 9) was higher than that in the other groups, and new epithelium completely covered the injured area. In contrast, the rates of re-epithelialization in the vehicle (53.87 ± 23.86%, n = 9) and HPDA groups (58.24 ± 36.24%, n = 9) were only half that of the HPDAlR group, although new epithelium was also found in the RL-QN15 group (83.91 ± 33.27%, n = 9).

The most common sequela of scald is skin scar, which affects the appearance and joint function. If the burn is serious, sepsis, lung infection and acute kidney failure may also occur, which may be fatal [51]. Our results indicated that RL-QN15 had the ability to promote the healing of scalded skin injury. More importantly, the loading of HPDA significantly improved the healing ability of RL-QN15.

### HPDA increased the pro-healing activity of RL-QN15 against oral ulcers in rats

**Fig. 7:**
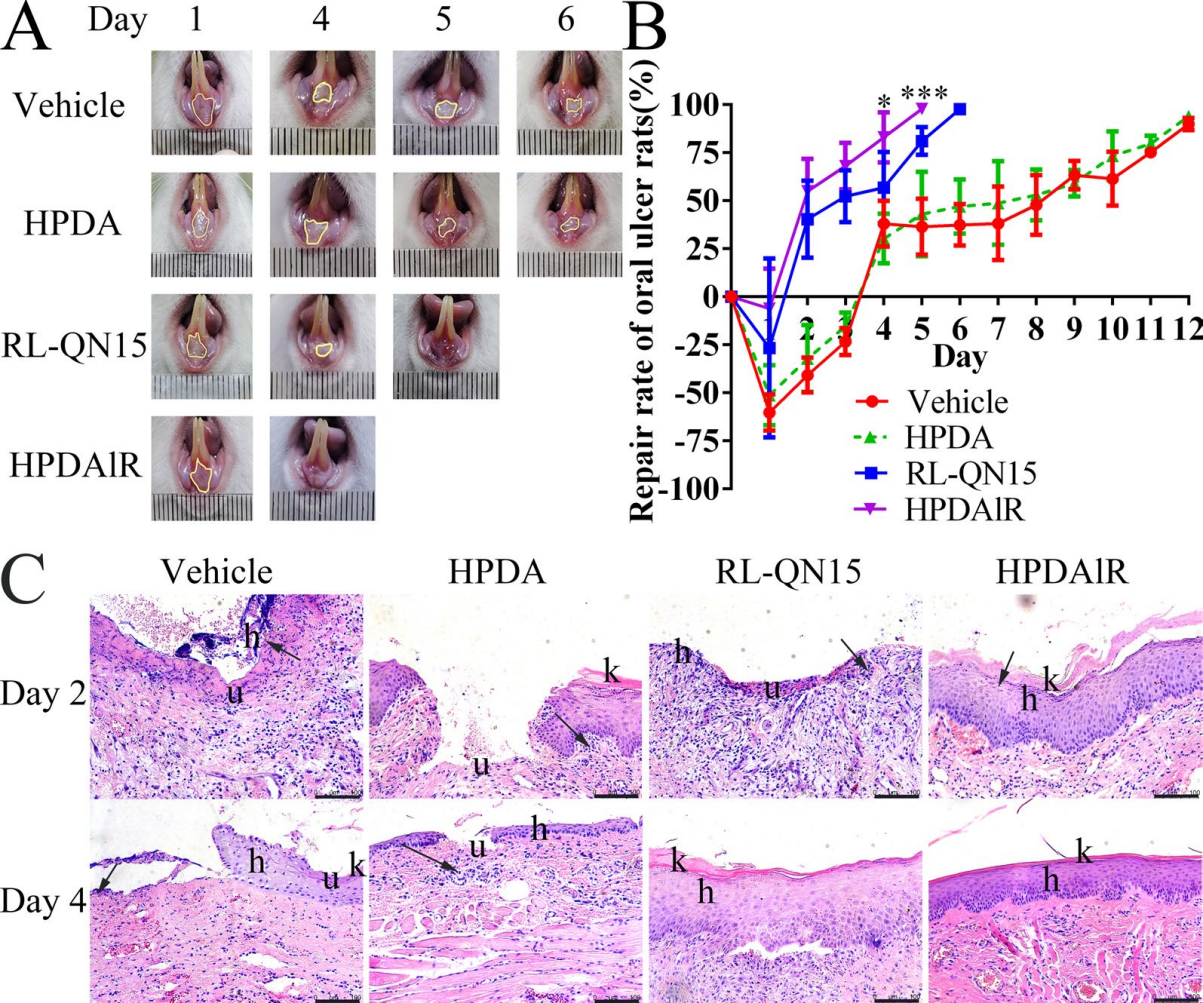
HPDA increased the pro-regenerative activity of RL-QN15 against oral ulcers in rat.** A** Representative images of oral ulcers on different postoperative days, showing effects of Vehicle (PBS), HPDA (0.1 mg/mL), RL-QN15 (1 nM), and HPDAlR groups. **B** Quantitative healing curves of ulcers treated with vehicle (PBS), HPDA, RL-QN15, and HPDAlR. Values were expressed as Mean ± SD (n = 10). Error bars represent SD. *P < 0.05, and ***P < 0.001 indicated statistically different from RL-QN15 group (Student *t*-test). **C** Representative images of histopathological changes in oral ulcers in vehicle (PBS), HPDA, RL-QN15, and HPDAlR groups on postoperative days 2 and 4. u, ulcerated oral epithelium. h, chronic hyperplastic epithelium. K, hyperkeratosis. Inflammatory cells were indicated by black arrows. Scale bar was shown by black line in bottom-right corner of images

Oral ulcers, a common oral mucosal disease, manifest periodically and are accompanied by burning pain. Ulcer tissue is prone to infection, inflammation, and tissue necrosis. There are many causes of oral ulcers, including immune disorders, drug stimulation, and bacterial infections [52]. Our previous research has proved the pro-regenerative effects of RL-QN15 against oral ulcers in rats [27], in the current research, we further to verify whether the load of HPDA could increase the pro-regenerative effects of RL-QN15 against oral ulcers in rats. Oral ulcer recovery in normal rats takes about 12 days, showed by our previous research [27], which was also verified in the current research, here, as shown in Fig. 7A and B, compared with vehicle, HPDA nanoparticles showed no positive effect against the healing of oral ulcers, as contrary, local application of RL-QN15 and HPDAlR accelerated the healing of oral ulcers. When treated with RL-QN15 (1 nM) or HPDAlR, it took 6 and 5 days, respectively, for all oral ulcers to heal completely. In our previous research, the total recovery of oral ulcers in 7 days required the treatment of RL-QN15 with 10 nM [27]. Thus, it might be reasonable to speculate that the therapeutic effects of HPDAlR were 10 times greater than RL-QN15 alone.

As shown in Fig. 7C, on postoperative day 2, oral ulcer formation and inflammatory cell accumulation were observed in the vehicle, HPDA, RL-QN15, and HPDAlR groups. With the progress of treatment, the therapeutic effects of RL-QN15 and HPDAlR became increasingly significant, and at postoperative day 5, the surface of the oral ulcer was completely covered by new oral mucosal epithelium and was considered to have healed. In contrast, significant oral ulcers with only partial mucosal epithelium were observed in the PBS and HPDA groups.

Periodic and repeated attacks of oral ulcers can have a serious impact on a patient’s work and life. Because of the complicated etiology and unknown pathogenesis of oral ulcers, there is no specific therapeutic drug in clinical practice. Severe patients usually receive local symptomatic treatment supplemented with systemic treatment, which can produce marked side effects and unsatisfactory outcomes [53]. Compared with the use of peptide alone, the HPDAlR used in this study improved the oral ulcer repair rate by nearly 20%, indicating that HPDAlR might be novel option for the treatment of oral ulcers.

### HPDA up-graded the pro-regenerative activity of RL-QN15 in acute full-thickness injured skin wounds in swine

Previous studies have shown that skin thickness in mice is less than 50 μm, while that in swine and humans is 70–140 μm and 50–120 μm, respectively [54]. In addition, given its similar structure to human skin, swine skin is often used as an ideal model for studying skin trauma, evaluating dermatology, and developing cosmetic drugs [54]. In the current research, the effects of HPDA and HPDAlR against acute full-thickness injured skin wounds on swine were also evaluated.

**Fig. 8:**
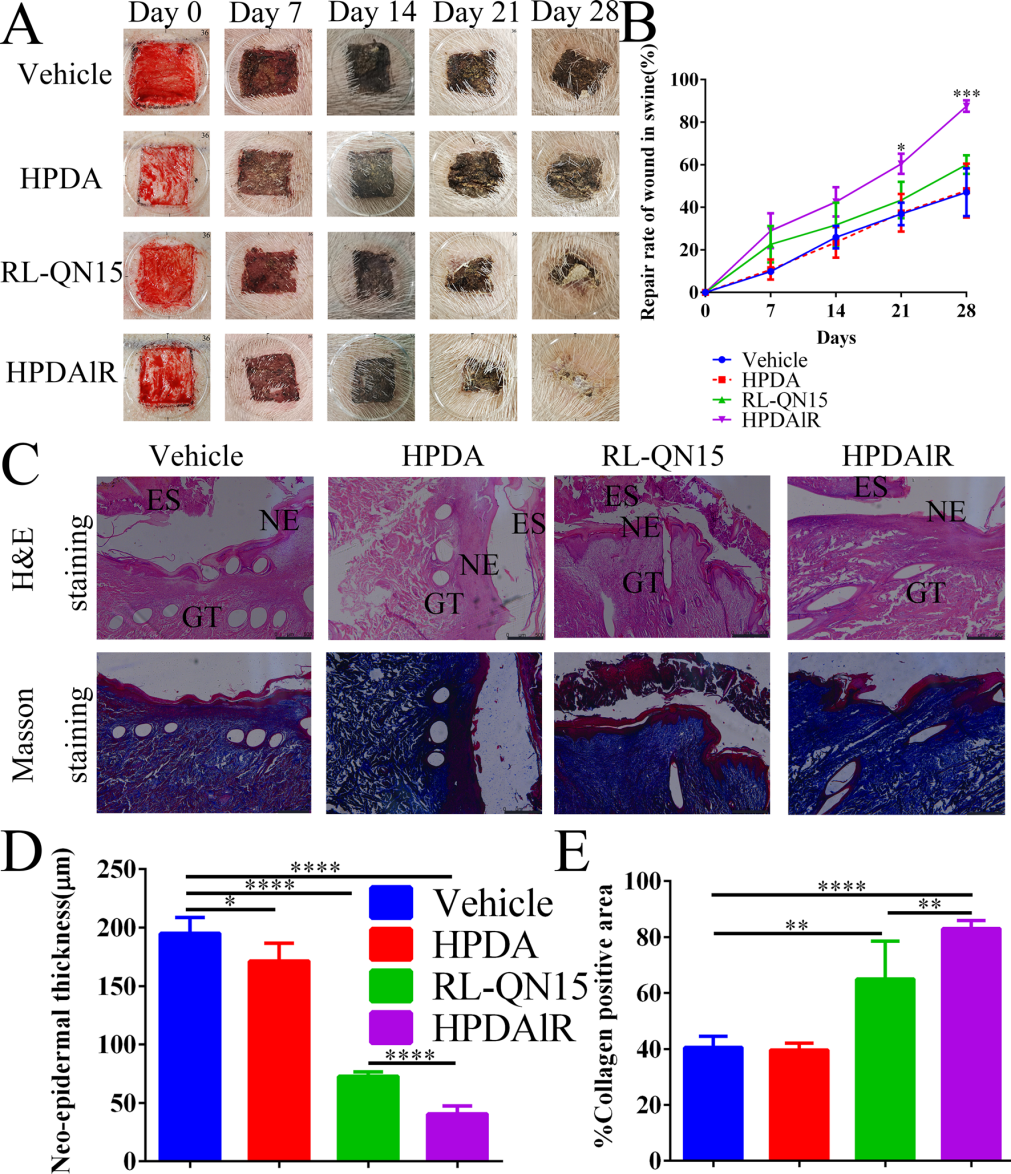
HPDA up-graded the pro-regenerative ability of RL-QN15 against acute full-thickness injured skin wounds in swine.** A** Representative images of full-thickness wounds on postoperative days 0, 7, 14, 21, and 28 topically treated with vehicle (PBS), HPDA (0.1 mg/mL), RL-QN15 (250 nM), and HPDA (0.1 mg/mL)lR(250 nM) groups. **B** Quantitative curves of curative effects of vehicle (PBS), HPDA, RL-QN15, and HPDAlR on full-thickness dorsal skin wounds in swine. Data were Mean ± SD from six wounds (n = 6). *P < 0.05, and *** *P* < 0.001 indicated statistical differences between two groups (Student *t*-test). **C** Representative images of skin wound sections stained with H&E and Masson’s trichrome on postoperative day 28 in vehicle (PBS), HPDA (0.1 mg/mL), RL-QN15 (250 nM), and HPDA (0.1 mg/mL)lR(250 nM) groups. NE, Neoepidermis; GT, Granulation tissue; ES, Eschar. **D–E** Quantitative analysis of neo-epidermal thickness and collagen positive area on postoperative day 28, respectively. Data were Mean ± SD from six wounds (n = 6). *P < 0.05, ** *P* < 0.01, ***P < 0.001, and **** *P* < 0.0001 indicated statistical differences between two groups (Student *t*-test)

As shown in Fig. 8A and B, both PBS and HPDA treatment did not promote swine wound healing, whereas RL-QN15 and HPDAlR treatment significantly promoted swine wound healing. On postoperative days 7, 14, 21, and 28, the average healing rates of the vehicle group were 9.85 ± 1.09%, 25.95 ± 6.12%, 36.87 ± 6.09%, and 47.06 ± 7.36%, respectively (n = 6), and those for the HPDA group were 10.74 ± 5.24%, 23.55 ± 7.61%, 37.39 ± 10.00%, and 47.83 ± 14.56% (n = 6), respectively. In contrast, on postoperative days 7, 14, 21, and 28, the average healing rates in the RL-QN15 group were 22.61 ± 9.34%, 31.68 ± 11.66%, 43.38 ± 9.82%, and 60.05 ± 5.02% (n = 6), respectively, which were ~ 1.28 times that of the vehicle group. Thus, RL-QN15 showed pro-regenerative potential in the full-thickness injured skin model in swine. In comparison, the healing rate in the HPDAlR group was significantly higher, with an average rate of 87.52 ± 2.74% (n = 6) on postoperative day 28, which was 1.86 and 1.46 times higher than that in the vehicle and RL-QN15 groups, respectively (Fig. 8B).

On postoperative day 28, the swine skin wounds were sampled for H&E and Masson staining. Histological analysis showed more neovascularization and hair follicles in the HPDAlR group compared with the other groups (Fig. 8C). In addition, as shown in Fig. 8D and E, the thickness of the new epidermis was 195.03 ± 20.3 μm in the vehicle group and 171.37 ± 10.91 μm (n = 6) in HPDA group. However, new epidermis was significantly thinner in the RL-QN15 (72.74 ± 4.87 μm) and HPDAlR groups (40.56 ± 11.18 μm, n = 6). These results indicated that RL-QN15 not only accelerates wound healing, but also effectively alleviated the increase in skin thickness, which was beneficial for the repair of scars. According to the Masson trichrome staining results, there was no significant difference in collagen content between the vehicle (40.52 ± 5.87%, n = 6) and HPDA groups (39.64 ± 3.2%, n = 6). However, collagen content in the HPDAlR group (82.96 ± 5.13%, n = 6) was 1.3 times higher than that in the RL-QN15 group alone (64.98 ± 16.21%, n = 6).

## Conclusions

In the current research, the prohealing peptide RL-QN15 was successfully loaded onto the HPDA nanoparticles hence HPDAlR was prepared. Our results showed that HPDAlR significantly enhance the pro-regenerative potency of RL-QN15, the underlying molecular mechanisms might be attributed to that through the satisfactory loading and slow-releasing efficacy of HPDAlR, the ability of RL-QN15 to selectively regulate the release of healing-involved cytokines from macrophages, hence, HPDAlR showed much more potent potential to accelerate the healing of keratinocyte scratch in vitro, the acute full-thickness injured skin wounds on mice and swine, scald on mice and oral ulcers on rat. Our research highlighted the strategy using HPDA nanoparticles to loading peptide with prohealing activity represented a novel intervention for the skin wounds and thus nanocomposites of HPDAlR hold great potential to be a promising pro-regenerative therapeutics.

Although the pro-regenerative abilities of RL-QN15 had been greatly improved, but there were still some aspects that need to be explored in terms of its performance and mechanisms. In theory, the structural–functional relationship of RL-QN15 awaited further explored, in the hope that shorter mutants of RL-QN15 with increased abilities were obtained, hence the cost would be sharply decreased. Besides, considering the clinical practical application situation, HPDAlR should be combined into hydrogel or surgical dressing, which would be performed in the following research. In addition, the effects of HPDAlR on the non-healing or chronic wounds should also be evaluated in the future research.

## Materials and methods

### Chemicals and reagents

All chemical reagents were of analytical grade and were used as received unless otherwise stated. Both 3-hydroxytyramine hydrochloride (dopamine hydrochloride), tris (hydroxymethyl) aminomethane (Trizma®base, ≥ 99.8%) and indocyanine green (ICG) were purchased from Sigma-Aldrich (USA) and used as received. Monodisperse polystyrene (PS) nanoparticles (50-nm diameter) were purchased from the Shanghai Hugebio Technology Company (China). Absolute ethanol and trichloromethane were bought from Sinopharm Chemical Reagent Co., Ltd. (China). The peptide RL-QN15 with purity higher than 95% used in this study were commercially synthesized and provided by Bioyeargene Biotechnology Co. Ltd. (Wuhan, China).

### Animals and ethics statement

Male Kunming mice (18–22 g, 4 weeks old), male BALB/C (nu/nu) nude mice (20–25 g, 8 weeks old) and Sprague–Dawley (SD) rats (180–220 g, 8–10 weeks old) were purchased from Hunan Slack Jingda Experimental Animal Co. Ltd. (Hunan, China). Diannan small era swine (10–15 kg) were obtained from the Experimental Animal Center of Kunming Medical University and were fasted before operation. The animals were acclimated to the laboratory for at least 7 days before use in the experiments. All animal care and handling procedures were approved and conducted in accordance with the requirements of the Ethics Committee of Kunming Medical University (kmmu2021743).

### The synthesis of HPDA nanoparticles

The HPDA particles were synthesized according to previous work [31, 32, 55]. In brief, dopamine aqueous solution (2 mg/mL) was prepared by dissolving dopamine (0.2 g) in 100 mL of Tris–HCl buffer solution (10 mM, pH 8.5). The PS powder (0.1 g) was dispersed in the dopamine solution by sonication, and magnetically stirred at room temperature for 24 h. After that, core–shell structured PS/PDA composite nanospheres were formed. These nanospheres were separated with a centrifuge (10 000 rpm, 15 min) at room temperature, washed in ethanol several times, and then dried in a vacuum oven at room temperature for 24 h. Finally, the PS/PDA composite nanospheres were further treated in trichloromethane to remove the PS templates and form hollow PDA nanospheres. The colloidal hollow PDA nanospheres were collected by centrifugation (10 000 rpm, 15 min) at room temperature and washed with ethanol several times. The HPDA nanoparticles were dried in a vacuum for 24 h, then re-dispersed in phosphate-buffered saline (PBS) for further use.

### The preparation of HPDAlR

The HPDAlR were prepared according to previously reported protocols [41]. Briefly, after the HPDA nanoparticles were adequately dissolved in PBS by an ultrasonic device (VCX750, Nanjing Xinchen Biotechnology Co., Ltd, China), the peptide RL-QN15 was incubated with the decentralized HPDA nanoparticles for 18 h at 4 °C, then the mixture was centrifuged (10 000*g*, 5 min, 4 ℃) and the supernatants were discarded and the rests were considered as HPDA nanoparticles loaded with RL-QN15 (HPDAlR). In addition, the successful loading of RL-QN15 onto the HPDA nanoparticles were confirmed by both Fourier transform infrared spectroscopy (FTIR, NICOLET-IS10, Thermo Scientific, USA) and an X-ray photoelectron spectroscopy (XPS, K-Alpha XPS spectrometer, Thermo Fisher Scientific, USA) equipped with Al Kα radiation (hv = 1 486.6 eV).

### Characterization of HPDA nanoparticles and HPDAlR

The morphology and structure of HPDA were investigated by transmission electron microscopy (TEM, Talos F200s, FEI, USA). Elemental information of HPDA was characterized by energy-dispersive X-ray spectroscopy (EDX, Super-X EDS Detector, FEI, USA). In addition, the surface area and pore size distribution of HPDA were determined by a Brunauere Emmette Teller (BET, ASAP 2460 3.01, Micromeritics, USA) and the specific surface area (S_BET_) and mean pore diameter were calculated by using the BET theory [56]. Total pore volume was calculated based on liquid nitrogen adsorbed at P/P0 = 0.99. The Barrett–Joyner–Halenda (BJH) theory was used to determine the pore size distribution from the nitrogen adsorption values.

### The preparation of HPDA and HPDAlR labeled with ICG

The ICG-labeled HPDA and HPDAlR were prepared as per previous report [57]. Briefly, Both ICG solution (dissolved in PBS, 1 mg/mL, 5 mL) and HPDA (dissolved in PBS, 20 mg/mL, 1 mL) were completely dispersed under ultrasonic condition for 5 min (VCX750, Nanjing Xinchen Biotechnology Co., Ltd, China). Then HPDA solution with volume of 25 μL were added into the ICG solution and stirred for 2 h by a magnetic stirrer (V055107, Thermo Scientific, USA) and centrifuged for 3 times (10 000*g*, 5 min, 4 ℃) to remove excessive ICG and the sediments were kept and hence ICG-labeled HPDA was obtained. Then, ICG-labeled HPDA was re-dispersed in PBS (0.1 mg/mL) and incubated with RL-QN15 (1 nM) for 18 h at 4 °C to obtain ICG-labeled HPDAlR.

### The determination of loading and slow-releasing efficiency of HPDA nanoparticles against RL-QN15

The loading efficiency of HPDA against RL-QN15 were determined according to the previous report with some modifications [58]. Typically, RL-QN15 was dissolved in PBS solution and the mixed solution’s relative concentration was determined via the absorbance at 220 nm determined by a spectrophotometry (NanoDrop One, Thermo, USA). PBS solution containing HPDA (0.5 mg) and RL-QN15 (100 nmol) with a volume of 5 mL was stirred at 4 °C for 24 h to form a nanocomposite solution, then centrifuged (10 000*g*, 5 min, 4 ℃), and the resulting supernatant was taken at various time-points (2, 4, 6, 8, 10, 12, 16, 20, and 24 h) to determine the relative concentration of the unloaded peptide RL-QN15. The loading efficiency of HPDA nanoparticles against RL-QN15 was calculated according to the equation: Loading efficiency (%) = [C (0) − C (2, 4, 6, 8, 10, 12, 16, 20, 24)] / C (0) × 100, where C (0) represented the initial concentration, and C (2, 4, 6, 8, 10, 12, 16, 20, and 24) represented the concentrations at 2, 4, 6, 8, 10, 12, 16, 20, and 24 h, respectively [19, 30].

To measure the releasing efficiency of HPDAlR, the HPDAlR were re-suspended in PBS at 37 ℃ and then when at different time (1, 2, 4, 6, 8, 12, 16, 20, and 24 h), the supernatants were collected by centrifugation (10 000*g*, 5 min, 4 ℃) for the determination of peptide concentration. The releasing efficiency of RL-QN15 from HPDAlR was determined quantitatively using the equation: Releasing efficiency (%) = C (t)/C (0) × 100, where C (t) represents the content of RL-QN15 in the supernatant at different time points and C (0) represents the initial content of RL-QN15 in the HPDAlR [59].

### The determination of the toxicity of both HPDA nanoparticles and HPDAlR

The toxicity of both HPDA nanoparticles and HPDAlR were evaluated at cellular and animal levels. The cytotoxicity of HPDA nanoparticles and HPDAlR against immortalized human keratinocytes (HaCaT) and macrophage Raw264.7 were determined by a CCK8 assay kit (Dojindo, Japan) [27]. HaCaT were cultured in DMEM/F12 (BI, Israel) supplemented with 10% (v/v) fetal bovine serum (FBS, BI, Israel) and antibiotics (100 units/ml penicillin and 100 units/ml streptomycin) at 37 °C in a humidified atmosphere of 5% CO_2_.The HaCaT or RAW264.7 cells were seeded into 96-well plates (~ 3 × 10^3^ cells/100 µL per well) and cultured in 5% CO_2_ at 37℃ for 12 h. Then 200 µL of vehicle (serum-free medium), HPDA (0.01 mg/mL), RL-QN15 (1 nM) and HPDA (0.01 mg/mL) lR(1 nM) were added to each well and incubated for another 12 h. Before 10 µL of CCK8 solution was added to each well, the cells were cautiously washed with PBS. After 4 h of incubation, the absorbance at 450 nm of each well was measured by a microplate reader.

In addition, the cytotoxicity of HPDAlR towards HaCaT cells were further evaluated by Live/Dead Cell Viability Assay [19]. Briefly, HaCaT cells were seeded into12-well plates at a density of 3 × 10^5^ cells/1 mL per well for 12 h. Next, the 1 mL of vehicle (serum-free medium), HPDA, RL-QN15 or HPDAlR were added into the well and incubated at 37 ℃ for 12 h continuously. Then the cells were cautiously washed with PBS by two times and analyzed by a Calcein-AM/PI Double Stain Kit (Beyotime, Shanghai, China) according to the standard protocols provided by the manufacture. Finally, the fluorescence imaging of live and dead HaCaT were imaged and analyzed by a confocal laser scanning fluorescence microscopy (Axio observer Z1, ZEISS, Germany).

The toxicity of HPDA and HPDAlR against mice with full-thickness injured dorsal skin wounds were evaluated. Briefly, when at the 8^th^ day, mice with full-thickness injured dorsal skin wounds treated with PBS, HPDA (0.1 mg/mL), RL-QN15 (1 nM) and HPDAlR were sacrificed and the main organs (heart, liver, spleen, left lung, and left kidney) were sampled, fixed in 4% neutral buffered formalin and processed routinely into paraffin, sectioned into slices of 4–5 µm in thickness for hematoxylin and eosin (H&E) staining and then observed and pictured by a digital microscope (Leica DM4 B, Leica, Germany) [19].

### Cellular scratch healing assay against keratinocytes

HaCaT were cultured in DMEM/F12 (BI, Israel) supplemented with 10% (v/v) fetal bovine serum (FBS, BI, Israel) and antibiotics (100 units/ml penicillin and 100 units/ml streptomycin) at 37 °C in a humidified atmosphere of 5% CO_2_. The effects of vehicle (PBS), HPDA (0.01 mg/mL), RL-QN15 (1 nM), and HPDA (0.01 mg/mL)lR(1 nM) on the healing of HaCaT cell scratch were determined exactly according to our previous research [27].

### The determination of contents of cytokines released from macrophage RAW264.7

Mouse macrophage RAW264.7 were cultured in DMEM/High glucose (BI, Israel) supplemented with 10% (v/v) FBS (BI, Israel) and antibiotics (100 units/ml penicillin and 100 units/ml streptomycin) at 37 °C in a humidified atmosphere of 5% CO_2_. The effects of samples (vehicle, HPDA (0.01 mg/mL), RL-QN15 (1 nM), and HPDAlR) on the release of cytokines (TNF-α, TGF-β1, IL-1β, IL-6, and VEGF) from RAW264.7 cell were determined exactly according to our prior research by ELISA kits (NeoBioscience, Shanghai, China) [27].

### Acute full-thickness injured dorsal skin wounds model on mice

Adult male Kunming mice (n = 48, 20–25 g) were selected after feeding for a week and then anesthetized with 1% pentobarbital sodium, and dorsal hair was removed by an electric shaver to expose the skin, which was then cleaned with 75% alcohol. Two standardized round whole-cortical wounds (8 × 8 mm) were formed on the dorsal of each Kunming mouse [3]. The mice were then placed singly in comfortable cages with a heater until they awoke from anesthesia. 45 Kunming mice were randomly numbered and divided into three groups. In the first group (15 mice), left-side wounds were treated with PBS and right-side wounds were treated with RL-QN15 (1 nM). In the second group (15 mice), left-side wounds were treated with HPDA (0.1 mg/mL) and right-side wounds were treated with the HPDA (0.1 mg/mL)lR(1 nM). In the third group (15 mice), left-side wounds were treated with PBS (seven mice) or HPDA (0.1 mg/mL) (eight mice), while the right-side wounds in all 15 mice were treated with HPDA (0.2 mg/mL)lR(1 nM). All wounds were topically treated with different samples twice a day (20 μL each time). In addition, images were collected and recorded on days 0, 2, 4, 6, and 8. Wound area (percentage of residual wound area to initial wound area) was assessed using ImageJ software (NIH, USA), and wound healing rates were quantified using GraphPad Prism software. Hematoxylin and eosin (H&E) staining was used for histological analysis of wound tissue on postoperative days 4 and 8.

In addition, when on the 4th and 8th days of post-operation, the mice were sacrificed and tissues of the center of the wounds were sampled, weighted and then homogenized in ice-cold PBS (1:9, weight/volume), and centrifuged at 12,000*g* for 20 min at 4 °C. The supernatants were collected and TGF-β1 level were analyzed by using ELISA kits (NeoBioscience, Shanghai, China).

### The biodistribution and clearance of HPDA and HPDAlR

The biodistribution and clearance of HPDA and HPDAlR which were labeled with ICG and administrated by applied topically to Kunming mice skin wounds or injected into abdominal cavity of nude mice (0.1 mg/mL, 20 μL), respectively, were analyzed by a Bio-Real in vivo imaging system. The mice were anesthetized with isoflurane and then the fluorescent images, which represented the biodistribution and clearance of HPDA and HPDAlR, were scanned and pictured by a Bio-Real in vivo imaging system (IVIS Lumina Series III, PerkinElmer, USA) at predetermined time (0.5, 2, 4, 8, 12, 24 and 48 h) with excitation wavelength of 780 nm and emission wavelength of 831 nm [60].

### Scald skin wounds model on mice

Adult male Kunming mice (n = 30, 25–30 g) were selected after one week of feeding. The mice were anesthetized with 1% pentobarbital sodium and the dorsal hair was removed by an electric shaver to expose the skin, residual microvilli on the dorsal were cleaned with depilatory cream, followed by 75% alcohol for disinfection. Two burn wounds were established on the dorsal skins of each mouse [61]. The experimental wounds were induced using a 5-mL centrifuge tube (inner diameter 12 mm), in which the base was cut off and flattened and the interior was filled with boiling water with a 2-mL syringe. The device was pressed onto the dorsal area of the treated mice for 15 s to form a skin scald injury. The 30 mice were randomly divided into two groups. In the first group (15 mice), left-side wounds were treated with PBS and right-side wounds were treated with RL-QN15 (1 nM). In the second group (15 mice), left-side wounds were treated with HPDA (0.1 mg/mL) and right-side wounds were treated with HPDA (0.1 mg/mL)lR(1 nM). All wounds were treated twice a day (20 μL each time) and images were collected on days 1, 4, 8, and 12. Wound area (percentage of recuperative wound area to original wound area) were estimated from the images using ImageJ software (NIH, USA). In addition, wound tissue on postoperative days 8 and 12 was analyzed histologically. All results were analyzed quantitatively by GraphPad software.

### Rat oral ulcer model

Adult male SD rats (n = 40, 180–200 g) were anesthetized with an intraperitoneal injection of 30% pentobarbital sodium solution (100 μL/100 g). A cylindrical glass tube (6-mm diameter) filled with cotton balls soaked with 15 μL of 30% glacial acetic acid was oppressed on the gum of the lower lip for 20 s, leading to a uniform and circular ulcer [27]. The 40 rats were randomly divided into four groups. Each group of 10 rats was treated with PBS, RL-QN15, HPDA (0.1 mg/mL), and HPDA (0.1 mg/mL)lR(1 nM), respectively. All wounds were treated twice a day (50 μL each time) and images were collected on days 1, 4, 5, and 6. ImageJ software (NIH, USA) was used to estimate wound area (percentage of recuperative wound area to original wound area), and GraphPad Prism was used to quantify the wound healing rate. Wound tissue was further sliced on postoperative days 2 and 4 and examined histologically by H&E staining.

### Swine full-thickness dorsal injury mode

Two male swine were fed to approximately 15–20 kg. Before the study, the animals were fasted for 12 h, although were given free access to water. An intramuscular injection of chlorpromazine (0.15 mL) was used to sedate the animals. After 5 min, an intravenous injection of 3% sodium pentobarbital was given in the ear (1 mL/kg). Dorsal hair was then shaved, and skin was washed with PBS and disinfected with 75% alcohol. Using a 25 × 25 mm stencil mark, a wound pattern was drawn at 50-mm intervals on the dorsal skins skin for forming wounds [62]. Sterile No. 22 scalpel blades were used to cut the fat layer in the marked area. Finally, 24 wounds of the same size were formed on the dorsal of the swine. The 24 wounds were divided into four groups, which were twice daily treated with PBS, HPDA (0.1 mg/mL), RL-QN15 (250 nM), or HPDA (0.1 mg/mL)lR(250 nM). Wound condition was assessed and ImageJ was employed to measure wound area (percentage of recuperative wound area to original wound area). GraphPad Prism was used to analyze the wound healing rate. Both H&E and Masson trichrome staining were used for histological analysis of wound tissue sections on postoperative day 28.

### Histological analysis

All tissue samples were immobilized in 4% paraformaldehyde for 24 h, dehydrated, transparentized, and embedded in paraffin [24]. For histological analysis, paraffin-containing tissue blocks were cut into 4–5-μm slices and stained with H&E or Masson trichrome. All sections were imaged under a Primovert microscope (Zeiss, Germany) to observe regenerated epidermis and granulation tissue. To evaluate new epidermal and granulation tissue thickness after skin trauma, we randomly measured 30 areas in the images and analyzed them using ImageJ software (NIH, USA).

## Supplementary Information

**Additional file 1: Table S1.** Surface areas, pore diameter and pore volume of HPDA nanoparticles. **Figure S1.** Biodistribution and clearance of HPDA and HPDAlR.

## Data Availability

The datasets used and analyzed during the current study can be obtained from the corresponding author on reasonable request.

## Abbreviations

HPDA: Hollow polydopamine
HPDAlR: HPDA nanoparticles loading with RL-QN15
TGF-β1: Transforming growth factor-β1
TNF-α: Tumor necrosis factor-α
IL-1β: Interleukin-1β
TEM: Transmission electron microscopy
EDX: Energy-dispersive X-ray spectroscopy
FTIR: Fourier transform infrared spectroscopy
XPS: X-ray photoelectron spectroscopy
BET: Brunauer–Emmett–Teller
EGF: Endothelial growth factor
PBS: Phosphate-buffered saline
Calcein-AM: Calcein acetoxymethyl
PI: Propidium iodide
IL-6: Interleukin-6
VEGF: Vascular endothelial growth factor
LPS: Lipopolysaccharide
H&E: Hematoxylin–eosin
ICG: Indocyanine green
PS: Polystyrene
SD: Sprague–Dawley
FBS: Fetal bovine serum
