# Supplementary material for: Hollow polydopamine nanoparticles loading with peptide RL-QN15: a new pro-regenerative therapeutic agent for skin wounds
Source: J Nanobiotechnology. 2021 Oct 2;19:304. doi: 10.1186/s12951-021-01049-2 (PMC8487533; doi:10.1186/s12951-021-01049-2)
Supplement: Supplementary file 1 — Additional file 1: Table S1. Surface areas, pore diameter and pore volume of HPDA nanoparticles. Figure S1. Biodistribution and clearance of HPDA and HPDAlR. [file 12951_2021_1049_MOESM1_ESM.docx]

**Table S1. Surface areas, pore diameter and pore volume of HPDA nanoparticles.**

| **Sample S_BET_^a^m^2^/g** |  | **BJH Pore Diameter (nm)** | | **Pore volume (cm^3^/g)** |
| --- | --- | --- | --- | --- |
| **HPDA 39.1667** |  | **27.2621** | **0.252455** | |

**^a^ Surface area.**

**
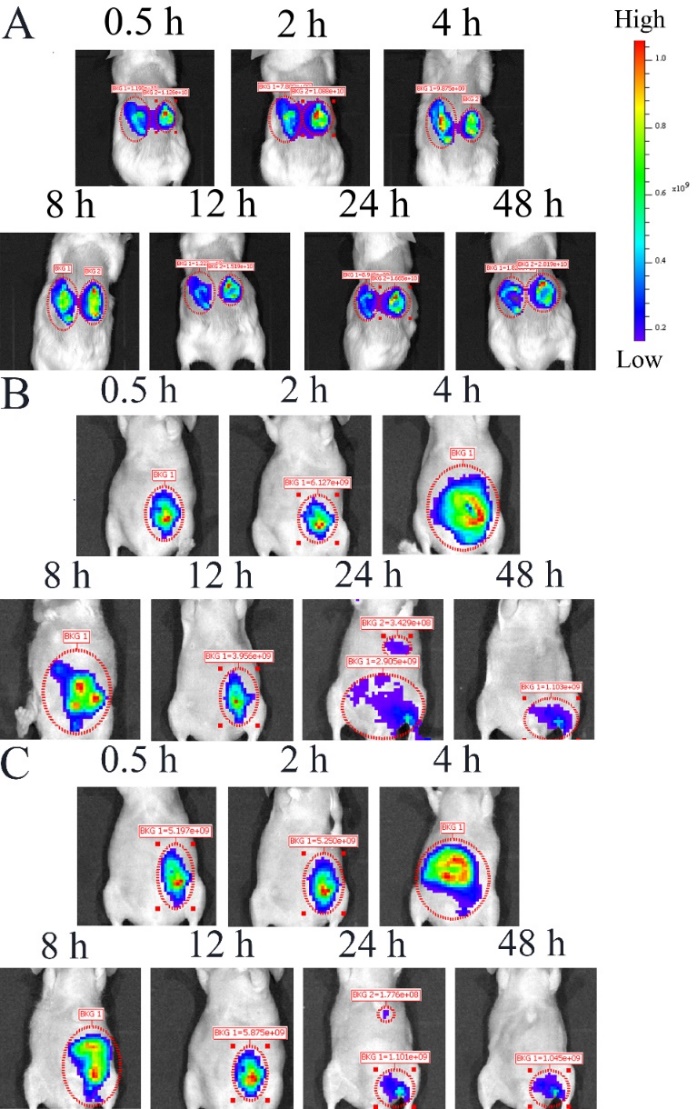
**

**Table S1. Surface areas, pore diameter and pore volume of HPDA nanoparticles. Figure S1.** **Biodistribution and clearance of HPDA and HPDAlR.**

**A.** *In vivo* fluorescence imaging of Kunming mice smeared with HPDA and HPDAlR by recording ICG fluorescence at indicated time (0.5, 2, 4, 8, 12, 24, 48 h).

**B.** *In vivo* fluorescence imaging of nude mice intraperitoneal injected with HPDA by recording ICG fluorescence at indicated time (0.5, 2, 4, 8, 12, 24, 48 h).

**C.** *In vivo* fluorescence imaging of nude mice intraperitoneal injected with HPDAlR by recording ICG fluorescence at indicated time (0.5, 2, 4, 8, 12, 24, 48 h).
